# Population and Unemployment

**Published:** 1924-10

**Authors:** Colonel Maurice

**Affiliations:** C.M.G.


### POPULATION AND UNEMPLOYMENT.

### THE PURPOSE OF THE UNIVERSE.

By COLONEL MAURICE, C.M.G.

UNEMPLOYMENT is the problem of to-day, of to-morrow, and of morrows to come. Not all the plans hitherto put together and contrived are enough to give work to one half of the workless—to give more work, not profitable, income-producing work. That seems beyond expectation. Much of the suggested employment, far from being profitable will entail, if it be given, a charge for decades to come upon the wealth-making, wealth-saving and wealth-owning part of the community. The wages of useful work, interest, the reward of the self-denial which is creating, and has created, capital, are to be taxed now, and during a long future, to pay for work not profit-giving, put in hand only to give excuse to pay money.

### Road Improvements and Housing Schemes.

Consider the schemes. The chief is road-making and road-improvement—exceeding pleasant for some of the road-users. Motor traffic may go with great speed and use larger vehicles, and more fast-travelling conveyances can take the road. That, it is said, will improve trade, enable larger profits and more wages. Perhaps; but will the larger profits and the more wages equal the bill to pay? There are housing schemes. To them there can be no objection. Good houses are urgently necessary. There are thousands upon thousands of young married men and women who cannot afford to build themselves houses or to pay 5 per cent. on the cost of building them of the standard of comfort and convenience they desire. So people who deny themselves to create capital refuse to create it in the form of houses. The State now has more than a million potential wealth producers that none will provide with raw materials and tools wherewith to produce wealth; they are the unemployed. It is not easy to see how the thousand a day increase of the population is going to help in the production of wealth. It is possible that, later on, they also will find none to provide them with materials and machinery and that they may swell the numbers of the unemployed potential wealth producers. They must be wealth consumers whether or not they produce.

### Reducing Food-bearing Land.

Of course, they will stimulate State house production, for if, each year, half a million or so of extra people join the community, after a time, at five to a family, they require a hundred thousand more houses year by year. So the unemployed can be put to work on State housing schemes in increasing numbers, for an infinite series of decades, given space. There's a hitch. Every house takes up an area of land, much of it farm-land. Along the new and widened roads one scheme plans that two or three hundred feet of breadth of land is to be taken. On and along this breadth houses are to be built. Now, suppose two hundred and ten feet of depth be taken. Then, every seventy yards of road, so far as the road runs through an agricultural district, takes out of cultivation about two and a half acres to the mile. Beyond this breadth, back roads parallel to the main ones, with communicating roads, are talked about, that still more building sites may be available not far off the arterial roads. Many two hundred and fifty-acre farms may be lost to food production under such schemes, and many agricultural labourers may become superfluous.

### Population and National Greatness.

The food produced in Britain will be less, but what matter! The producers of exchangeable wealth must work the harder and exchange their products for more and more of foreign foods and share with the road makers, the house builders, the canal diggers and our rapidly increasing population! We are told that we may congratulate ourselves upon this increase because a stationary population is a sign of national decline, whereas a rapidly increasing population is an index of national greatness. Is that certainly and always right? We have it by rote and have accepted it by reason of repetition; but there seems to be growing an uneasy feeling that possibly a land may have too many people, a suspicion that after a certain number per acre has been reached, not necessarily the same number in every country, increase is excess. There is questioning on every side and strong propaganda of a nature that would have caused all but universal horror a quarter of a century ago, but that now is received calmly. May it not be that the State has not yet explored the root cause of the evil?

### The Growth of Urbanisation.

Now is it certain that times will so improve and that other countries will so keenly compete for our manufactures that our men now unemployed and the yearly increase of our population will all, or most of them, be busily at work to meet demand and will be receiving from abroad, in return for the wealth they make, food and luxuries up to a high standard of living? In other lands also peoples are increasing. The increases of the populations are flowing from the villages into large towns. Towns are places where food is not produced, but is obtained and consumed in exchange for things made. These other lands abroad are also being urbanised. Their peoples may be pressed to use the things their own townsmen make rather than those ours produce, even though their men make them not so well as ours.

### Emigration.

If this be so, our excess population must emigrate and fill the vast unfilled spaces of the earth. Where are these spaces? They are not so many nor so very great. Maps are deceptive. Moreover, how many of our people have aptitude for the cultivation of great vacant spaces, strength, courage and skill to coax a living from bare soil? These men are of white race, of temperate climes, therefore they can live and work and breed only on a small part of the surface of the earth, and that part is filling fast. The United States are no longer eager to receive emigrants, because their own population begins to press on their space. Canada is willing to take selected persons, and desires only those who can and will till the soil in a bleak land of great cold far north none but the hardiest can endure. Men from crowded streets are appalled by silence and loneliness. Australia asks for more population, so long as it is of a sort that will not compete with organised labour in her towns. She asks for people to do what the Australian will not do, that is, remain in the bush and toil on the land, a pleasant land and fruitful where water can be found. Like to Canada, Australia will adopt none likely to become a charge on the community. She must be allowed to pick and choose only of the best. Africa is available for none but the comparatively wealthy. There is a native population question there. New Zealand wants but few emigrants. Thus it comes to this, that some of the best of our surplus population, for the present, are welcomed, perhaps not very warmly, in some other lands. None of the worst and very few of the middling are acceptable.

### What is the Remedy ?

An increase of population which leads to large drafts of the best material leaving the homeland, and to all the worst and most of the middling staying in it to breed more increase, cannot by any known law of heredity be a cause of national greatness. If, as it may be, our unemployment is at root due to over-population, and if it is being made worse by increase, and if that increase cannot be employed or got rid of by emigration, and if the population is in danger of deterioration because only the best may settle abroad and all the worst must remain behind, what is the remedy, if remedy there be ? That is for the community, led by its statesmen, to consider and discover. From the nature of the problem it would seem that it must be some form of limitation of births complemented by improvement of the quality of the births allowed—a grave, wise, considered control based on knowledge and the study of heredity, perhaps a new religion, grimly altruistic, calling for individual sacrifice grievous to be borne ; longings suppressed for good of the kind, with for reward the satisfaction of duty done at cost ; national and racial sentiment and strong sentimentality sternly crushed. This may mean the perishing of old beliefs, but the springing up of a strong new faith, a conviction that the Cause of the Origin of the Universe intended, if we may presume to assume intention, all creatures to be happy and to evolve towards perfection.

---

### Whitehall Bars Radiators.

An interesting announcement has been made by the Ministry of Health in a letter received by the Honiton Guardians. They were proposing to instal a system of gas radiators in the sick wards and the nursery of the institution, and in due course submitted their proposal to Whitehall for approval. A letter has been received stating that the experience of their inspectors is against the use of radiators in rooms intended for human occupation. Whether the Ministry considers that radiators heated by hot water are equally undesirable does not appear.

